# Supplementary material for: Detection of Apparent Early Rabies Infection by LN34 Pan-Lyssavirus Real-Time RT-PCR Assay in Pennsylvania
Source: Viruses. 2022 Aug 23;14(9):1845. doi: 10.3390/v14091845 (PMC9504839; doi:10.3390/v14091845)
Supplement: Supplementary file 1 [file viruses-14-01845-s001.zip › S1_Text.pdf]

Supplemental Materials and Methods: Direct Fluorescent Antibody (DFA) Test

PABOL

Brain tissue representing a full transverse cross section of brain stem and three lobes of cerebellum and/or hippocampi were minced together. These brain tissue preparations were used to make impressions onto ThermoScientific Gold Seal cat 3032 FA slides, Erie Scientific LLC. For each sample, two impressions per slide were prepared in duplicate. No buffer or external moisture was added during the tissue preparation. Samples were dried for 15-30 minutes at room temperature and then fixed for at least one hour in acetone at -20°C. DFA test was then performed using two rabies-specific monoclonal antibody conjugates: EMD Millipore 5100 and EMD Millipore 6500. Non-rabies monoclonal antibody conjugate specificity control, EMD Millipore 5102 was used for both samples, and Fujirebio 800-092 anti-rabies conjugate was used only for the 2019 sample. The conjugate preparations were filtered into a sterile 2 mL tube using Pall Corporation filters Ref # 4604 Acrodisc 13mm diameter 0.45µm porosity Supor Membrane. For slide preparation, 30 to 35 µL of the filtered conjugate was added per well. The slides were incubated in a moist chamber at 37°C for 30 minutes and then rinsed with PBS and deionized water. Slides were viewed on either or both Nikon Eclipse Ci and Nikon Eclipse 50i using 20x Apochromat 0.75 NA and 40x Fluorite 0.75 NA objectives both with a HBO 100/w3 light source. Scoring for intensity and distribution was based on observations and consultation between two readers using the standard 1-4 response scale with 1+ being lowest level response up to 4+ being the highest level. In both cases, samples were re-tested by DFA after positive rRT-PCR result. During re-testing, separate samples from cerebellum, brain stem, hippocampi, and rostral spinal cord were collected from the original frozen brain tissues. Brain impressions

were made directly from intact tissue including a complete transverse cross section of brain stem, representative tissue from three lobes of cerebellum and additional tissues (Table 2) using the staining protocol as described above. For this testing, custom slides (Cel-Line ThermoFisher Scientific Cat 30-226H-RED, 2 well 15mm HTC autoclavable) were utilized. Operators were not blinded during re-testing of samples.

CDC

Samples were tested according to the Protocol for Postmortem Diagnosis of Rabies in Animals by Direct Fluorescent Antibody Testing, A Minimum Standard for Rabies Diagnosis in the United States and Direct Fluorescent Antibody Test, WHO, Laboratory Techniques in Rabies [13, 16]. Brain impressions were made on to (Cel-Line Epredia Cat 30-31H-White, 3 well 11mm HTC autoclavable) slides directly from the tissues received and included a full cross section of brain stem and aliquots of the three lobes of cerebellum. For the 2019 juvenile raccoon, additional tissues (hippocampi, cortex) were tested per the request of PABOL. Brain impressions were dried for 15-30 minutes at room temperature, and were fixed in acetone for 1 hour at -20°C. Slides were then placed in a moist chamber, and 2 drops (100-150 µl) of working dilutions (prepared in PBS with Evans blue counterstain) of EMD Millipore 5100 and Fujirebio 800-092 FITC labeled anti-rabies monoclonal antibody conjugates, and EMD Millipore 5102 FITC non-rabies monoclonal antibody conjugate (specificity control, isotypes matching EMD Millipore 5100) were added directly to the impressions using a syringe equipped with a low protein binding 13 mm 0.45 µm filter (Pall, Ref # 4604 Acrodisc, Supor) per the reference protocol to remove any particulates and avoid non-specific fluorescence. Slides were incubated at 37°C for 30 minutes. After incubation, impressions were rinsed individually with a wash bottle containing PBS and each slide was placed in a separate container of PBS for 2 exchanges for 5

minutes each. Per the reference protocol no water rinse was used. Slides were removed directly from the PBS (to maintain tissue and antigen integrity), and the brain impressions were blotted on adsorbent paper and coverslips (Corning 24 x 50 mm #1) attached with low (20%) glycerol Tris-buffered mounting medium (EMD Millipore 5096). The impressions were examined by two readers for the presence of rabies virus antigen using a Zeiss AxioImager microscope equipped with HBO 100 light source (OSRAM 103/w2). All impressions were observed by both readers first with a 20x Apochromat lens 0.80 NA (for maximum detection intensity) and 40x Apochromat lens 0.95 NA when observing morphology only. The intensity and distribution of typical rabies-like inclusions were recorded for each impression. Operators were not blinded to the samples.
